# Design of an Interactive Virtual Reality System, InViRS, for Joint Attention Practice in Autistic Children

**DOI:** 10.1109/TNSRE.2021.3108351

**Published:** 2021-09-16

**Authors:** Ashwaq Z. Amat, Huan Zhao, Amy Swanson, Amy S. Weitlauf, Zachary Warren, Nilanjan Sarkar

**Affiliations:** Robotics and Autonomous Systems Laboratory, Department of Electrical Engineering and Computer Science, Vanderbilt University, Nashville, TN 37235 USA; Robotics and Autonomous Systems Laboratory, Department of Electrical Engineering and Computer Science, Vanderbilt University, Nashville, TN 37235 USA; Vanderbilt Kennedy Center, Treatment and Research Institute for Autism Spectrum Disorders, Nashville, TN 37203 USA; Department of Pediatrics, Vanderbilt University Medical Center, Treatment and Research Institute for Autism Spectrum Disorders, Nashville, TN 37203 USA; Department of Pediatrics, Vanderbilt University Medical Center, Treatment and Research Institute for Autism Spectrum Disorders, Nashville, TN 37203 USA; Department of Electrical Engineering and Computer Science and the Department of Mechanical Engineering, Vanderbilt University, Nashville, TN 37212 USA, and also with the Robotics and Autonomous Systems Laboratory, Vanderbilt University, Nashville, TN 37212 USA

**Keywords:** Intelligent system, autonomous systems, virtual reality, human computer interaction, gaze tracking, joint attention, Autism

## Abstract

Many children with Autism Spectrum Disorder (ASD) exhibit atypical gaze behaviors related to joint attention, a fundamental social-communication skill. Specifically, children with ASD show differences in the skills of gaze sharing and gaze following. In this work we present a novel virtual reality (VR)-based system, called InViRS, in which children with ASD play games allowing them to practice gaze sharing and gaze following. InViRS has three main design contributions: (i) a closed-loop joint attention paradigm with real-time tracking of the participant’s eye gaze and game performance measures, (ii) an assistive feedback mechanism that provides guidance and hints in real time, and (iii) a controller that adaptively changes the avatar’s gaze prompts according to the performance measures. Results from a pilot study to evaluate the feasibility of InViRS with 9 autistic^1^ children and 9 typically developing (TD) children offered preliminary support for the feasibility of successful gameplay as well as positive impacts on the targeted skills of gaze sharing and gaze following.

## I. Introduction

AUTISM spectrum disorder (ASD) affects approximately 1 in 54 children in the US [1] with significant associated costs [2]. Many children with ASD experience impairment in joint attention – a fundamental social skill that requires gaze sharing and gaze following with another person. Joint attention, which is different from simply making eye contact, is crucial to learning new information, knowledge exchange, and early language development [3]–[5]. Joint attention skills can be defined as the ability to coordinate one’s attention with another person towards an object or an event of interest [6]. There are two main components in joint attention: gaze sharing and gaze following. In gaze sharing, one is required to be aware of the other person’s gaze and intent to share information. In gaze following, which emerges after gaze sharing, one is required to shift one’s gaze and attention to the object or event being shared. Joint attention can be initiated by another person, which is known as response to joint attention (RJA) or can be initiated on their own, which is known as initiation of joint attention (IJA). Behavior-based interventions have shown promise in imparting joint attention skills in young children [7], [8], but their cost and trained personnel requirements limit their availability [7].

Although not posited as a replacement for skilled clinical care, technology-based interventions can complement and support behavioral intervention by increasing attention and learning in autistic individuals [9], many of whom show an affinity for technology [10]. Virtual reality (VR) based intervention, although not a substitute for human intervention, can provide a safe environment wherein autistic children can interact with a system to practice on their skills [11]. To assess engagement and response, VR can be integrated with peripheral sensors such as eye trackers and physiology sensors to provide measures of eye gaze [12] and physiological response [13], [14]. In recent years, VR-based joint attention studies have explored gaze perception, cognition, focus, and engagement in autistic individuals during joint attention interaction [15]–[18]. However, only a few studies [15], [16] have examined gaze sharing and gaze following specifically.

The primary contribution of this work is the design, development, and preliminary assessment of a novel **In**teractive **Vi**rtual **R**eality **S**ystem (InViRS), an adaptive game-based system for practicing core joint attention skills of gaze sharing and gaze following. In InViRS, a RJA paradigm initiated by a virtual avatar acts as an interaction partner that provides participants with gaze prompts through a closed-loop joint attention paradigm and real-time hints using continuous measurement of eye gaze and game performance. Rather than attempting to train individuals to make sustained eye contact, which many people with ASD describe as uncomfortable [19], [20], this system instead teaches them how to use another person’s gaze to gather important information about the environment as well as that person’s intentions and interests.

The current work substantially expands our previous conference paper [21] in terms of i) system augmentation, ii) introduction of an individualized adaptation model and iii) data from a pilot study. System augmentation included adding a new dimension to the avatar’s gaze prompts by manipulating the depth of the eye movements together with varying speed of the avatar’s gaze prompts and the inclusion of new region of interests on the avatar’s face to observe participants’ gaze fixation in a detailed manner. In addition, we present new results of a pilot study involving autistic and typically developing (TD) children.

The presented research contributes to the design of a real-time gaze detection algorithm, a task difficulty adjustment algorithm, an avatar controller that adjusts the avatar’s behaviors, and a supervisory controller that has embedded logic to coordinate the closed-loop interaction for individualized joint attention practice based on real-time measurement. Such a system itself is novel in this field and in our opinion, contributes towards the design of a new adaptive behavioral intervention system for ASD. Endowing InViRS with these abilities allows us to analyze RJA performance at the component level - gaze sharing and gaze following performances - in addition to overall RJA performance, a uniquely important contribution to this area of research, as the technologically facilitated ability to parse joint attention skills at a more granular level will potentially allow the development of targeted behavioral intervention. The remainder of the paper is organized as follows: Section 2 presents relevant literature reviews; Section 3 describes system design and architecture; and Sections 4 and 5 present the experimental setup and the results of the study, respectively. Finally Section 6 presents discussion on the potential and limitations of the current study.

## II. System Design

**Fig. 1.**
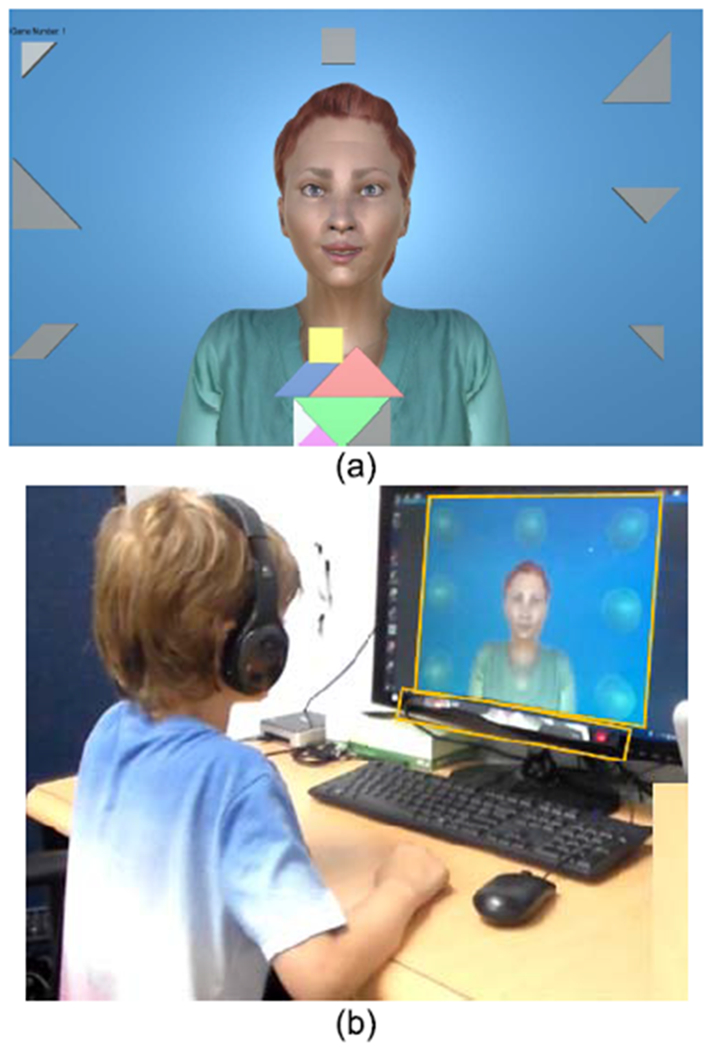
The virtual game environment. (a) Tangram puzzle game. (b) A participant playing the bubble popping game.

InViRS was developed as a game-based system through which children with ASD can practice the skills of gaze sharing and gaze following. Although InViRS is capable of delivering multiple game modes, in its current form, children play two different games with a virtual avatar: a Tangram Puzzle game, used for practice, and a Bubble Popping game, used for pre- and post-assessment (see Figures 1(a) and 1(b), respectively, and section II-A). Research shows that simple puzzle games are engaging for children with ASD [49]. We chose the Tangram puzzle game for joint attention practice in the hope that it would keep participants engaged. It was not too complex so as not to frustrate the participants, but at the same time had enough variation to keep the participants interested. We also wanted to choose a simple game for pre and post assessment that was both easy to control and visually interesting. The Bubble Popping game satisfied both these criteria. Both games were successfully used in our previous work with children with ASD [50], [51]. Each game involves systematic assessment of children’s eye gaze in response to scaffolded prompts, across varying difficulty levels. InViRS has several options to create individualized and adaptive interaction with the child: 1) provision of varying gaze prompts, 2) delivery of prompts and visual aids using the least-to-most (LTM) prompting mechanism, 3) an adaptive module that changes the avatar’s interaction level to match the participant’s performance, 4) variation in the speed of gaze prompts to actively probe participant’s ability to follow gaze, and 5) real-time computation of game performance.

### A. InViRS Games and Human-Computer Interaction

**Fig. 2.**
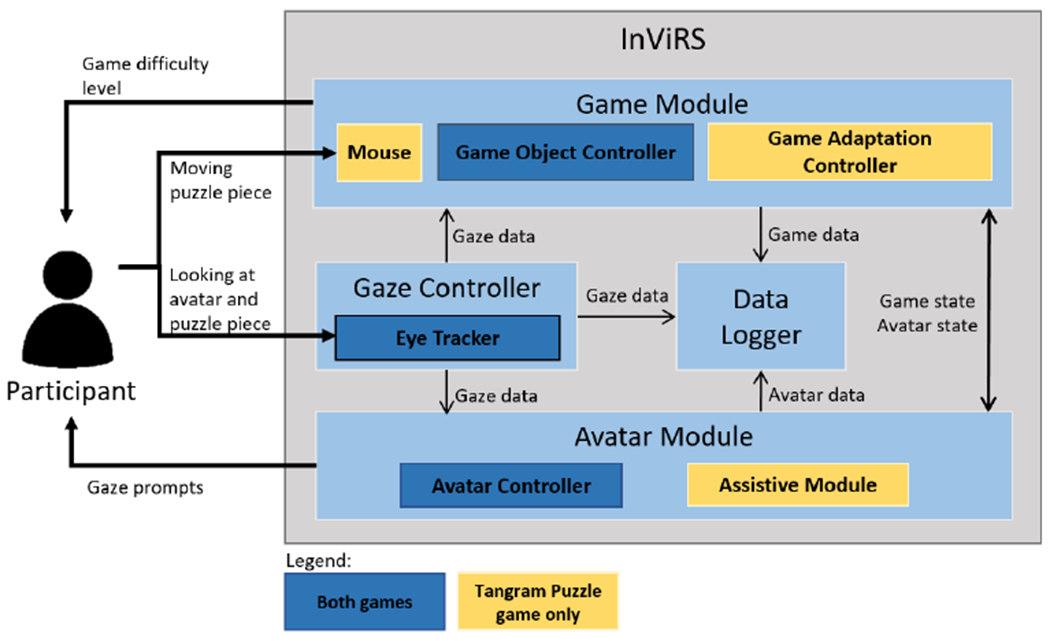
Human-computer Interaction block diagrams for InViRS. The game adaptation controller and the assistive module are not activated for the bubble popping game.

Figure 2 illustrates the interaction diagrams between the participant and InViRS. The eye tracker and mouse captured the participant’s gaze data in both games and puzzle pieces movement in the Tangram Puzzle game. The Gaze Controller i) sends gaze data to the Avatar Module to trigger the avatar’s gaze prompts, ii) updates the Game Module, and iii) logs the gaze data in the Data Logger. The Game Module manages the difficulty level of the game through the Game Adaptation Controller where difficulty level can be changed based on the gaze data, game states, and avatar states. The Assistive Module in the Avatar Module provides hints and assistance based on the participant’s performance.

Note that because of the structure of the Bubble Popping game, only the eye gaze data from the eye tracker are used to interact with the avatar and select the correct bubble to pop. Since there is no Assistive Module or Game Adaptation Controller in this game, the avatar’s gaze prompts and game difficulty level are increased continuously without any assistance or adaptive adjustments to the difficulty level.

#### 1) Gaze Sharing:

**Fig. 3.**
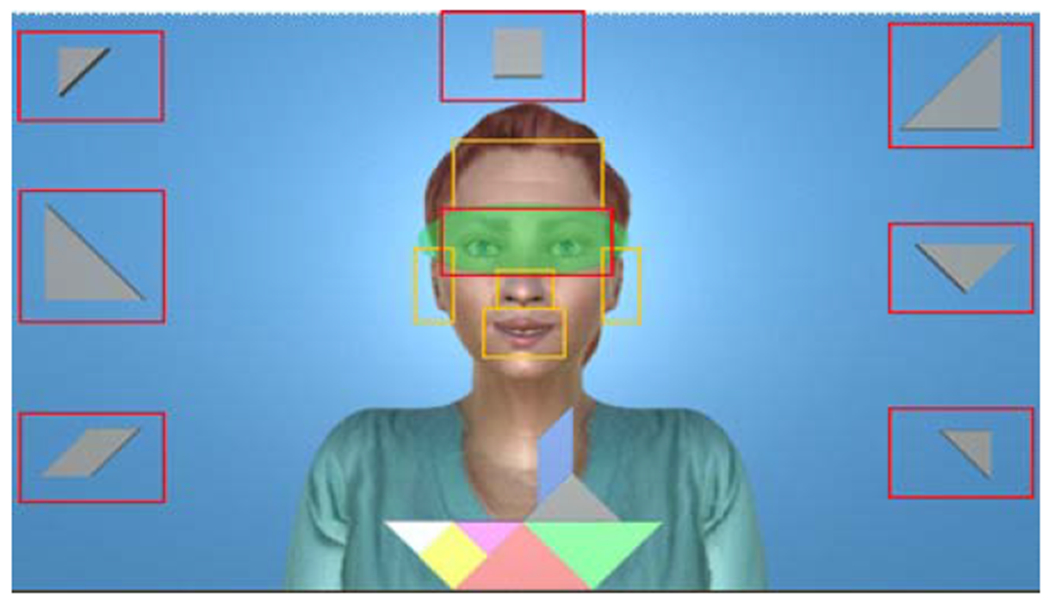
The ROIs for the Tangram puzzle game. Red boxes represent active ROIs and yellow boxes represent passive ROIs.

Within InViRS, gaze sharing is defined when a participant fixates their gaze on a predefined region around the avatar’s eye (Figure 3), and not necessarily directly on the avatar’s eyes. This was designed so that gaze sharing could be established without inducing the stress that may be evoked within individuals with ASD when they are forced to make direct, sustained eye contact [18], [19]. We chose a minimum duration for fixation of 200 ms based on the study presented by Rayner as a reasonable human gaze fixation characteristic [41]. When a gaze lasts more than 200 ms, the avatar will trigger the next prompt by shifting its gaze towards a game object (either at a puzzle piece in the Tangram Puzzle game or at a bubble in the Bubble Popping game).

We setup InViRS to wait for 30 seconds for a gaze to be registered on the avatar’s eye region before progressing to the next state. We chose 30 seconds in consultation with clinical psychologists specializing in ASD intervention as we wanted to give enough time for the children to receive the cue, process and respond to the avatar’s prompt. Longer waiting time might cause the children to lose focus and interest in the game. If participants did not look at the avatar’s eye region within 30 seconds, the system provided audio and visual cues. In the Tangram Puzzle game (practice), an audio cue in the form of 3 seconds of bell ringing was played and a visual cue of highlighting the avatar’s eye region was provided. In the Bubble Popping game (assessment), only the 3 seconds bell ringing audio cue was played if participants did not look. For both games, if no eye contact was made within 2 minutes, the game was terminated.

#### 2) Gaze Following:

As mentioned previously, after a participant successfully share their gaze with the avatar, InViRS triggers an event for the avatar to direct its gaze at a game object. The participant then needed to direct their gaze to the game object that was prompted to trigger the next event in InViRS.

In the Tangram Puzzle game, after the participant looked at the correct game object, the color of the object was revealed and the participant could move the puzzle piece to the target area using the mouse. If a participant did not look at the correct game object within 30 seconds, InViRS triggered assistive events from the Assistive Module to get the participant to look at the intended area. For example, the avatar would repeat the gaze prompt at a slower pace together with highlighting the puzzle piece it prompted. Details of the assistance for the Tangram Puzzle game is presented in II-E.

As for the Bubble Popping game, when the participant looked at the correct bubble, the bubble would pop and new bubbles will be generated. If no gaze was detected on the correct bubble within 30 seconds, no assistive events were triggered and the avatar proceeded to provide the next gaze prompt.

### B. Virtual Game Environment

**Fig. 4.**
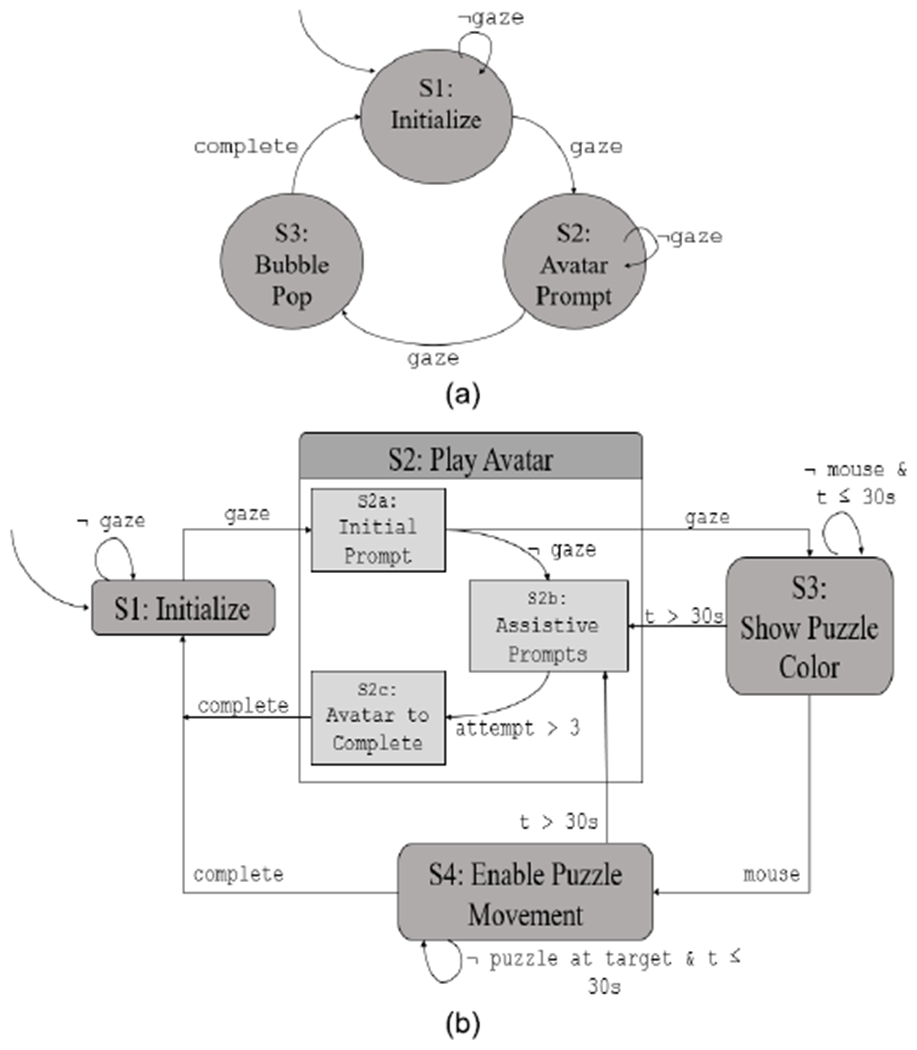
Finite state machines (FSM) for InViRS virtual environment. (a) FSM for the bubble popping game. (b) FSM for the Tangram puzzle game.

The virtual game environment was developed using Unity v5.6.1f1 [22], a widely utilized virtual game development tool. Both games in the virtual environment were developed as finite state machines (FSM). We defined a 5-tuple deterministic FSM as detailed in Table I. Figure 4 illustrates the FSMs for both games.

### C. Gaze Controller

In this study, we designed a controller that used eye tracking data from a Tobii EyeX [23] eye tracker in real-time to perform gaze analysis. The sampling frequency of the eye tracker is comparatively low, between 50-60 Hz, but is sufficient for use in this study, as the primary interest is on fixation data points rather than pupil diameter, saccades, and other fast-moving gaze points [24]. We used a Tobii-Unity development package [25] to: i) continuously collect gaze points during game play, and ii) register a gaze fixation on a predefined region when a gaze duration of approximately 200 ms [41] was measured. The gaze points that were collected in this controller were sent to the Data Logger to be recorded together with the time stamp and game state at that time.

Additionally, we defined several regions of interest (ROIs) in Unity to capture participant’s gaze on these areas. There were two categories of ROIs, active and passive, created for the objects and avatar in the games. The active ROIs were defined on the avatar’s eye region and all game objects in the games (puzzle pieces and bubbles). Taking into consideration the difficulty in autistic children to look directly at someone’s eye gaze [19], [20], we defined a rectangular region around the avatar’s eye to reduce discomfort when establishing gaze sharing. When a gaze was first detected on the avatar’s eye ROI, the controller would start a timer to measure the duration of the gaze. If the duration was more than 200 ms [41], the controller would trigger an event to the Avatar Module to indicate gaze sharing was initiated. If the duration of the gaze was less than 200 ms [41], the gaze would not trigger any event and the timer was reset before a new gaze was detected on the eye region again. The same algorithm was used when a gaze was detected on a game object ROI. If the gaze was detected on the correct game object for 200 ms, the controller would trigger an event to the Game Module to indicate that the correct game object was looked at.

As for the passive ROIs, five facial areas of the avatar were selected that included: the forehead, right ear, left ear, nose, and mouth. When a gaze was detected on a passive ROI, the controller would send the name, location and time stamp of the ROI to the Data Logger to be recorded. Figure 3 shows all the ROIs in the Tangram Puzzle game environment. The ROIs definitions are not limited to the objects in the Tangram Puzzle and Bubble Popping games and can be used in other VR environments that focus on gaze analysis or where non-verbal interaction is of interest.

### D. Avatar Controller

**Fig. 5.**
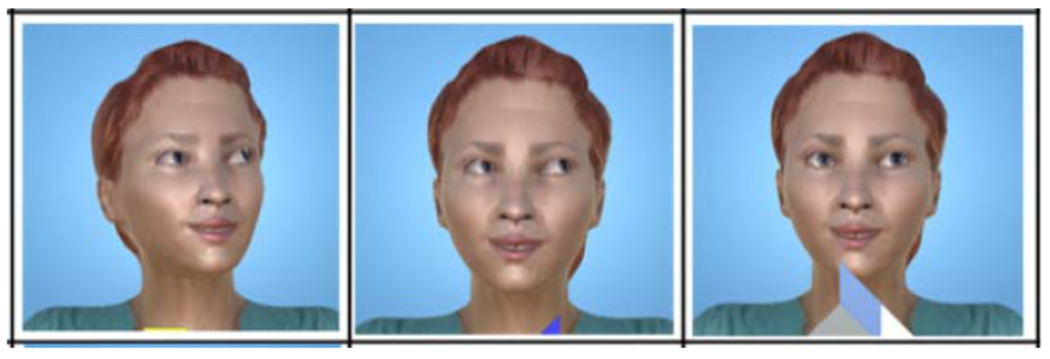
Example of avatar’s different eye gaze configurations in upward right direction. (a) Head movement together with eye movement, (b) Full eye movement, and (c) Minimal eye movement.

The design and animation of the avatar were accomplished using a 3D graphics application called Autodesk Maya [26]. The neutral facial expression for the avatar in this study was by design. Because the objective of this study was to evaluate the impact of a novel interactive virtual system on gaze sharing and gaze following, we chose a neutral expression to observe how participants responded to the eye gaze prompts without other factors, such as emotional valence, influencing the result. We customized the avatar’s head and eye movement such that the avatar could gaze in any direction to locate the relevant objects of the game. In this work, we created eight different gaze directions to correspond to the eight bubble pieces and seven tangram puzzle pieces. We also added different gaze prompt configurations for each gaze direction that consisted of animating the avatar’s head movement together with the eye movement, and manipulating the range of the movement of avatar’s eyeball from the center of the eye. Head movement has been shown to influence gaze following [27]–[29] eliciting faster response time when head and eye move congruently [30], [31]. As such, we used the head and eye movement together as the initial gaze prompts to represent an easy level. For the next gaze prompt difficulty level, we removed the head movement and only maintained the eye movements for gaze prompts. In this level, we had the avatar’s eye move from the center of the eye to the edge of the eye in the direction of the gaze prompt to represent maximum range of human eyeball movement [47]. For the third gaze prompt difficulty level, the avatar’s eyeball movement was reduced to 40% of the maximum movement range to create a subtle gaze prompt as judged by consensus of human observers. Figure 5 provides an example of the three gaze variations in the upper right direction.

The combination of using gaze prompts in varying direction, depth of eye movement and speed in this study demonstrates the flexibility of our avatar’s design that can be easily configured to support other gaze related implementations.

In both games, the gaze directions were randomly selected to avoid predictive behavior. For the Tangram Puzzle game, the different gaze prompt levels were evenly implemented as described in Table II. As for the Bubble Popping game, the gaze prompt level was kept at the second difficulty level and only the speed of the prompts was continuously increased.

### E. Game Adaptation Controller

The Game Adaption Controller is a part of Tangram Puzzle game that managed the change in the avatar’s interaction level with the participant based on participant’s performance. A rule-based adaptive algorithm was developed by using both game performance and gaze data as inputs to change i) the avatar’s gaze prompt level (as per Table II) and ii) the speed of the avatar’s gaze prompts. In addition to varying the avatar’s gaze prompt level, we also changed the speed of the avatar’s gaze prompts to make the game more challenging. The higher the speed of the gaze prompt, the harder it was for the participant to follow the gaze. For the Bubble Popping game, we did not use the Game Adaptation Module. The speed of the avatar’s gaze prompt in that game was increased at a constant rate in each prompt regardless of the participant’s performance in the Bubble Popping game.

**Fig. 6.**
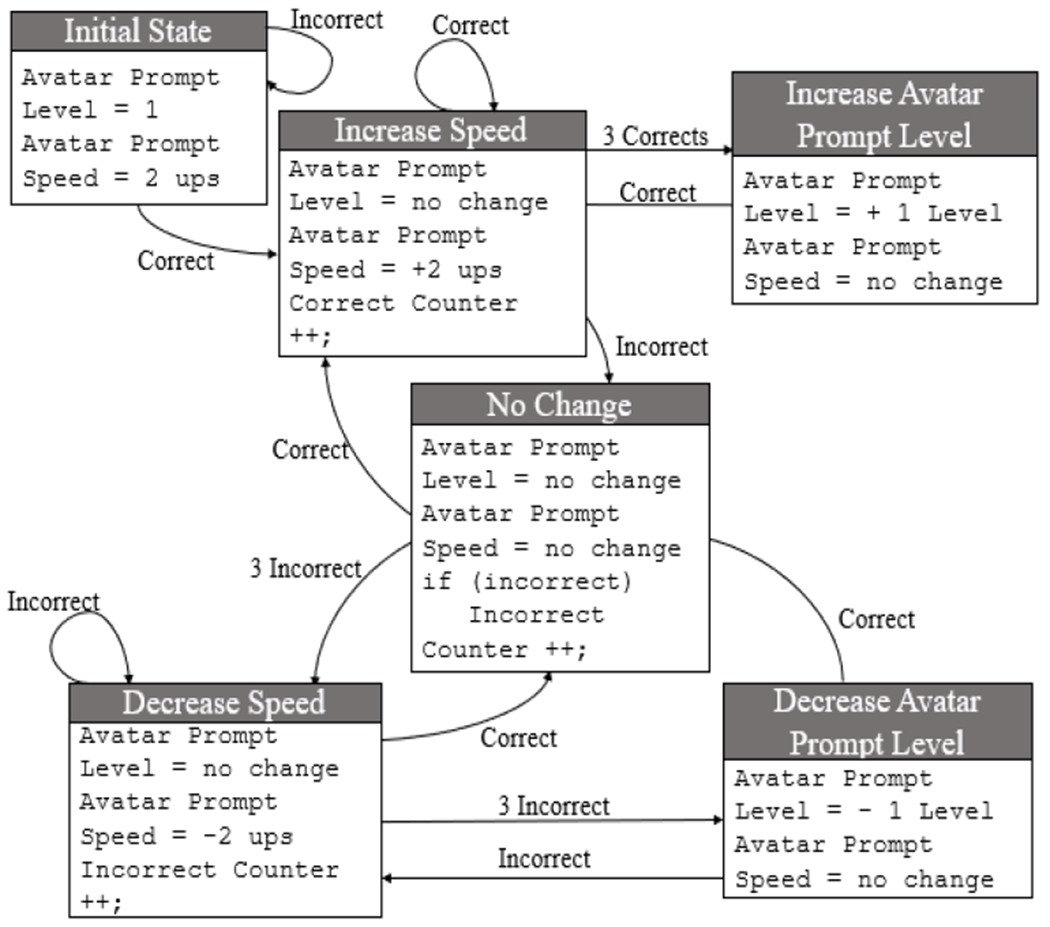
State diagram for game adaptation controller for Tangram puzzle game.

Figure 6 summarizes the adaptive algorithm. At the beginning of a Tangram Puzzle game, the gaze prompt level was set to Level 1 where the gaze prompt included the head movement together with eye gaze, while the speed of the avatar’s gaze prompt was set to a rate of 2 units per second (ups). When a participant correctly chose a puzzle piece that was prompted by the avatar, the subsequent speed of the avatar’s gaze prompt was increased at a constant rate of 2 ups. The speed remained the same when the participant failed to choose the correct puzzle piece. After three consecutive puzzle pieces were correctly selected, the gaze prompt level was increased such that the avatar’s gaze prompt was reduced to only eye gaze movements. Whereas, after three consecutive wrong attempts of choosing the corresponding puzzle pieces, the speed of the next gaze prompt was reduced by 2 ups. Then, if the participant continues to make three more consecutive incorrect selections, the avatar’s gaze prompt level was decreased to make the gaze prompts easier for the participant to follow and to provide opportunities for the participant to continuously strive and challenge their gaze following skills.

### F. Assistive Module

The Assistive Avatar Module was used only in the Tangram Puzzle game to assist the participants when they were unable to direct their gaze at the correct ROIs or in the intended direction. This module was not used in the Bubble Popping game.

The assistive avatar module used a least-to-most (LTM) prompting mechanism [32], which is widely used in intervention for children with ASD. The principle of LTM is to allow the learner the opportunity to independently execute the task with the least amount of prompting, which is then increased progressively depending on the need. The LTM mechanism has also been previously used to teach communication skills [33]–[35], and motor skills [36] in children with ASD. In this current study, LTM implies allowing the participant to interpret the avatar’s gaze prompt on their own before the avatar provides additional prompts leading the participant to the correct game object.

**Fig. 7.**
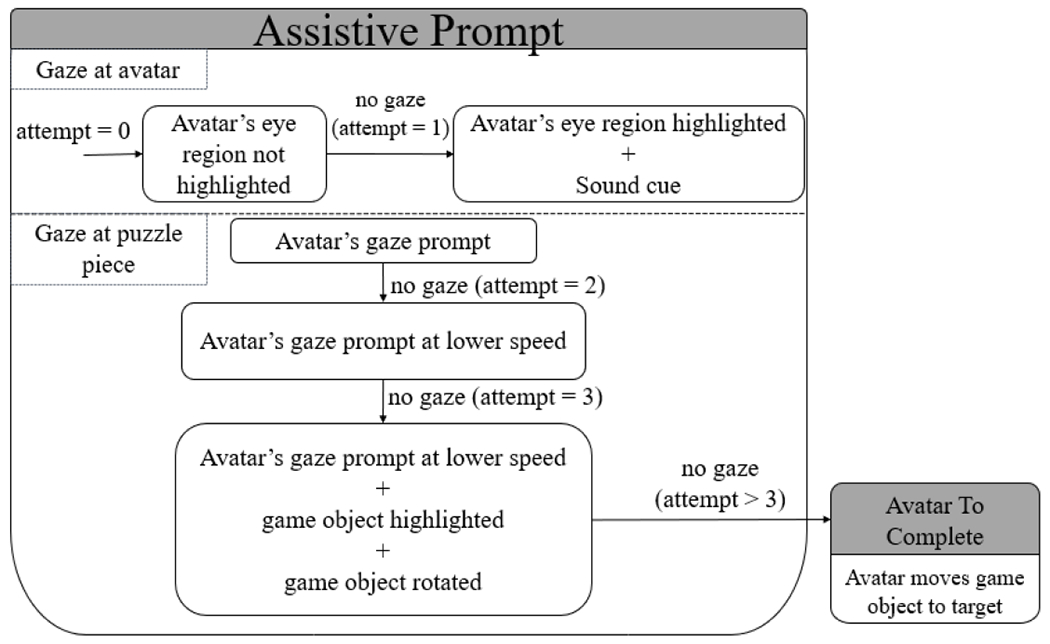
Flow chart of the avatar’s assistive prompt. Number of attempts increased when participant was unable to look at the correct place or game object.

Within our LTM design, we used both real-time gaze and current performance data as inputs to create a personalized assistance to the participants. For example, a participant performing at a higher gaze prompt level and higher prompt speed will receive a different assistive prompt compared to a participant performing at a lower gaze prompt level or prompt speed. This module supports individualized learning condition across different participants’ performance level. Figure 7 shows the progression of the assistive prompts for every unsuccessful attempt and Table III lists the assistance the avatar provided in order of number of attempts the participant made.

### G. Game Object Controller

The Game Object Controller manages the configuration of the game objects in both games. In the Bubble Popping game, this controller initialized the bubbles into their respective location in the virtual space. When a gaze event on the target bubble was received from the Gaze Controller, the Game Object Controller enabled the bubble to pop and waited 5 seconds before the bubble was regenerated at the same original location again. As for the Tangram Puzzle game, the controller initialized the puzzle pieces to their initial locations, set the appearance of each puzzle piece to zero color saturation (grayscale) and disabled their movements. When a gaze event on the target piece was received from the Gaze Controller, the Game Object Controller: i) displayed the color of the puzzle piece, ii) enabled movement of the puzzle piece, and iii) updated the movement of the puzzle piece to the target location. Once all the puzzle pieces were at the target location, the controller triggered an event to the game settings component to indicate the completion of the game and proceeded to the next game. This controller also tracks other game properties including the number of games played, duration of each game, points accumulated, and the number of assistances a participant used in each move.

### H. Data Logger

The data logger collected all the virtual environment data for real-time manipulation in the adaptive module and for offline data analysis. The real-time data used by the adaptive algorithm included participant’s game score, gaze ROIs, and avatar configurations.

## III. Experimental Design

We conducted a pilot study to evaluate the hypotheses that practicing in InViRS would be able to: i) improve gaze sharing in autistic children as indicated by increased in fixation frequency and duration on the eye region but not necessarily directly on the eye as compared to other facial features during interaction, and ii) improve gaze following skills in autistic children represented by improved game score. Additionally, we also wanted to compare game and gaze performance between ASD and TD participants to identify any meaningful differences. We administered a pre-test and post-test to assess changes in gaze fixation, gaze following, and performance measures after participating in practice session.

### A. Participants

We recruited a total of 18 children (9 children with ASD, 9 TD children) to participate in the study. The age range of the participants was between 7 and 13 years. Children with ASD were recruited from a large research registry maintained by the Vanderbilt Kennedy Center of children previously diagnosed with ASD by licensed clinical psychologists using standard diagnostic tools, such as the Autism Diagnostic Observation Schedule (ADOS) [37]. The TD children were recruited from the local community through regional advertisement.

To assess the current level of ASD symptoms of all participants and ensure baseline symptom differences between diagnostic groups, parents of all participants were asked to fill out the Social Communication Questionnaire (SCQ) [38] and the Social Responsiveness Scale, Second Edition (SRS-2) [39]. Both scales provide quantitative measures of observable characteristics of ASD via paper-and-pencil parent report. In this study, we used the SCQ Lifetime Total Score. This score ranges from 0 to 39, with a score above 15 indicative of likely ASD. For the SRS-2, participants received a Total Score and a T-score. A Total Score of 98 or a T-score value of 76 reflects high risk of ASD. Table IV presents the characteristics of the participants.

This study was approved by the Institutional Review Board at Vanderbilt University (IRB Number: 180047). Consents from the participants’ guardians and assents from the participants themselves were obtained before the experiment were conducted. A gift card was presented to participants at the conclusion of each visit.

### B. Protocol

The study consisted of three visits with 5 to 10 days between visits. In the first visit, the participants completed a pre-test which was the Bubble Popping game before starting the Tangram Puzzle practice game, and at the last visit, they completed another Bubble Popping game for post-test after finishing the last practice Tangram Puzzle game. The second visit was fully dedicated to practice with the Tangram Game. The order of each game was important since we needed to make sure that practice games were administered between the pre-test and post-test. At each visit, before starting any games, a participant’s eye gaze was calibrated on the Tobii EyeX eye tracker.

## IV. Results

Five performance metrics were defined to evaluate the hypotheses stated in Section III based on the results obtained from the Bubble Popping game in the pre- and post-tests. Table V lists the metrics together with a description of each metric. All statistical analyses were performed using MATLAB statistical computation functions. In this study, we calculated gaze fixation points in MATLAB using the EyeMMV toolkit [40].

### A. Overall Game Performance Measures

Game performance was measured using game score, time to complete the game, and the response time to each gaze prompt. First, on average, the autistic children improved their scores by 8 points in the post-test, which was closer to TD children’s game score in the pre-test. However, this improvement was not statistically significant. Meanwhile, the TD children did not show much improvement in the post-test compared to the pre-test, which may indicate that the TD children were already performing at their highest level in the pre-test because the game was not difficult for them. Next, we found statistically significant improvement in the time to complete the Bubble Popping game measure for autistic children (p = 0.0106). They improved on average by 1 minute and 20 seconds in the post-test, while the TD children spent 23 seconds less on average in the post-test. Lastly, autistic children showed improvement in the time to respond to the avatar’s gaze prompts measure, but the improvement was not statistically significant. On average they took 3.4 seconds to respond to the avatar’s gaze prompt in the pre-test, while in the post-test, they took on average 1.7 seconds to respond. Meanwhile, TD children spent almost the same time to respond in both pre-test and post-test, which were 1.6 seconds and 1.2 seconds, respectively. When looking at the effect size of the ASD participants, we observed a large effect size for the time to complete category, 1.333 which further support the statistically significant result. Medium effect sizes of 0.6711 and 0.7789 were observed for the game score and response time respectively, which indicate a meaningful increase in the ASD participants’ overall performance even though not all the categories were statistically significant. Note that for TD participants there were no statistically significant changes in all three categories even though the time to response had a medium effect size, 0.6702. Table VI presents the pre-test and post-test performance measures.

### B. Game Score Measures Based on Gaze Prompt Speed

**Fig. 8.**
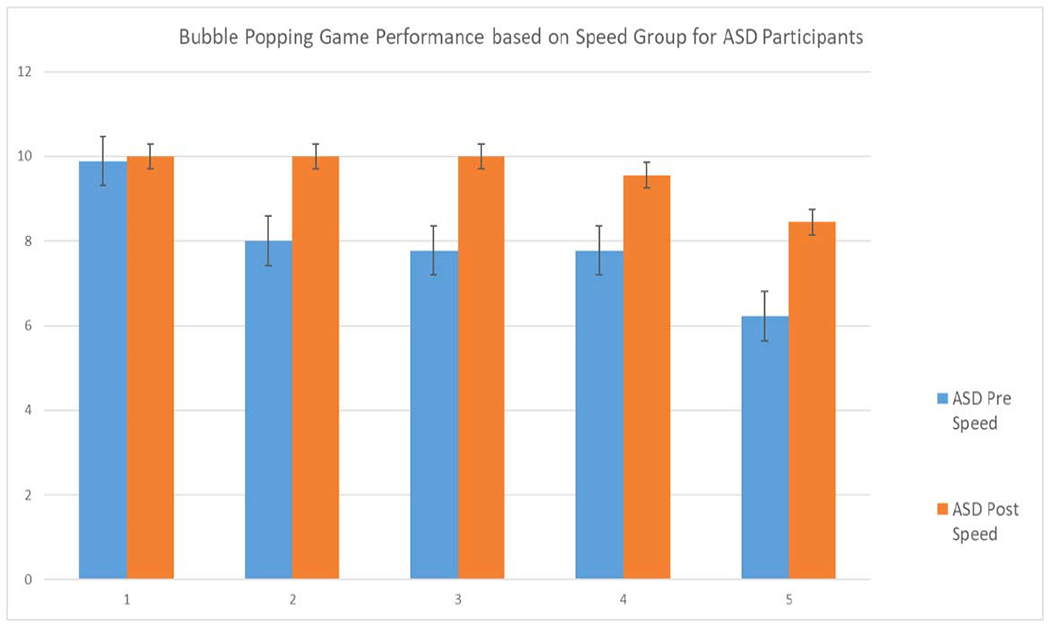
Performance comparison based on different speed grouping in pre and post-test for autistic participants in bubble popping game.

As mentioned in II-E, the speed of the avatar’s gaze prompt in the Bubble Popping game was increased by 2 ups each time the avatar provided a gaze prompt. Since the increment of the speed of gaze prompt in each turn was too small to be meaningfully analyzed individually, the avatar gaze prompt speed was clustered into five speed groups with a speed range of 10 ups in each cluster. For each group, the maximum score was 10 points. Figure 8 shows the performance in each speed group for both ASD and TD participants.

Table VII presents the results of statistical analysis using a t-test to compare the performance based on the different speed groups in the pre-test and post-test. The improvement in the performance was statistically significant for children with ASD (p = 0.0139). In the pre-test, the children with ASD were unable to keep up with the increase in speed of the avatar’s gaze prompt as shown by their scores progressively declining from Speed Groups 1 to 5. However, in the post-test, the children with ASD achieved maximum possible scores in Speed Groups 1 to 3. For Speed Groups 4 and 5, their post-test performances were significantly better than their pre-test performances although they did not achieve the maximum possible scores. TD children continuously received maximum scores in Speed Groups 1-4 in both pre- and post-tests with minimal improvement in post-test for Speed Group 5. Again, consistent with the findings in the previous analysis of game performance, the result suggested that TD children were already performing at their highest level in all speed groups.

### C. Gaze Fixation

Gaze fixation was calculated from the defined ROI gaze points and gaze durations in MATLAB using one of the functions called “*fixation_detection.m*” available on EyeMMV toolkit [40]. The function used two spatial parameters and one temporal parameter. The first spatial parameter, *t*1, was used to initialize a fixation cluster. The second spatial parameter, *t*2, was used to establish consistency in the cluster by removing gaze points that were outside the threshold of the second spatial parameter. The temporal parameter defined the minimum duration for fixation. Any fixation cluster with a duration smaller than the defined value was not considered as fixation and was removed. The selection of these spatial and temporal parameters was based on the type of task that was carried out. In our analysis, we choose *t*1 to be 1° of visual view and a minimum duration for fixation of 200 ms based on the study presented by Rayner [41] on reasonable human gaze fixation characteristic. As for *t*2, the threshold value was generated by the function by calculating the standard deviation from the fixation cluster.

To better understand the distribution of the participants’ fixation on the avatar’s face, we grouped the fixation points based on the ROI on the eye region and ROIs on other facial region. To get the fixation metrics for these ROIs, we ran the EyeMMV function for gaze points of each ROI separately. For example, to get the number of fixation points on avatar’s eye region, we used gaze points corresponding only to the avatar’s eye region, and to get the number of fixation points on other facial region of the avatar, we added the gaze points from the five passive ROIs; forehead, right ear, left ear, nose and mouth (as explained in II-D and in Figure 3). Table VIII represents the total fixation points on the avatar’s face and normalized fixation on the avatar’s eye region and other facial features.

The normalized result represents the ratio of the fixation points on the eye region to the fixation points on other facial features on the avatar’s face. There was a statistically significant increase (p = 0.0056) in the total fixation points on the avatar’s face region for children with ASD. However, there was almost no change in the total fixation points on the avatar’s face for the TD children with low effect sizes that indicated trivial differences in the TD eye gaze fixation.

## V. Discussion

We designed a novel VR gaze system, InViRS, to assess and teach skills related to two core features of joint attention: gaze sharing and gaze following in children with ASD. When designing the modules for InViRS, we wanted InViRS to accommodate the diverse learning abilities of autistic individuals since ASD is a spectrum disorder. Taking this into consideration, we designed and implemented the Game Adaptation Controller and the Assistive Avatar Module. The real-time use of eye gaze and game performance data in the Game Adaptation Controller created a personalized learning experience for children with ASD. Using the same real-time data in a supervisory logic embedded within the Avatar Assistive Module allowed InViRS to provide individualized hints or assistance when users were unable to progress in the tangram puzzle game.

We have successfully completed a pilot study using InViRS. In this study, children with ASD and TD children completed avatar-initiated RJA prompts in two games, one designed as a pre and post-test evaluation (Bubble Popping game) and one designed to allow real-time assistance and difficulty modification to prompt skill acquisition (Tangram Puzzle game). Gaze sharing was established by the avatar waiting for the participant to look its eye region before shifting its gaze toward the target. Gaze following was measured through the ability of the participant to correctly look at the object that was targeted by the avatar.

Based on the results and analysis presented above, we believe that this system has the potential to help children with ASD interpret important communicative gaze-based information as part of social interactions. Regarding gaze following, the overall performance of children with ASD improved as based on their higher game scores and shorter response times after practice with InViRS. This replicate other findings in the literature indicating that adaptive systems can enhance the learning experiences of people with ASD [42]. Regarding gaze sharing, children with ASD looked more frequently at the avatar’s eye region in the post-test as demonstrated by an increase in the ratio of fixation on the avatar’s ROI compared to other facial ROIs. This suggests that the assistive mechanism (LTM) embedded in the practice Tangram Puzzle games positively encourages the children with ASD to share their gaze with the avatar. This is consistent with the work [43], [44] supporting the use of a VR-system to assist individuals with ASD in shifting their attention to the desired object or event of interest. Results also suggest that the children with ASD learned that the avatar’s gaze communicated important non-verbal information with regard to the direction that they need to follow, as they spent less time looking for non-verbal prompts from other facial ROIs and more frequently directed their gaze at the avatar’s eye ROI over time. However, even after gaze sharing was established, gaze following was still challenging, especially when the gaze prompt was quickly administered.

We also found important and persistent between-group differences based upon the speed with which gaze prompts were administered. Participants with ASD showed significant improvement in their performance in all speed groups. This statistically significant improvement indicated that InViRS was able to help children with ASD to adapt and respond to the changes in gaze prompts speed. However, relative to TD participants, it was harder for participants with ASD to correctly follow the avatar’s gaze when it was quickly administered, even after they knew to look at the avatar’s eye ROI. Looking at the pre-test results presented based on the different speed groups, participants with ASD scored relatively low in the higher speed group while TD participants showed consistently high performance across all speed groups. Furthermore, increasing the speed of the gaze prompts also encouraged the participants to respond to each gaze prompt faster. Faster response time to gaze prompts could indicate a more efficient joint attention ability. As previously reported in [44], [45], response time in a joint attention prompt were correlated with verbal intelligence [45] and ability to process social information [44]. It is also interesting to report that in the highest speed group, both ASD and TD participants did not receive full score, which could indicate that the avatar’s gaze prompt speed in the highest speed group was hard to process.

The promising results of the current study further support InViRS as a system capable of tracking game data in varying configurations, accumulating game performance measures, adaptively changing the difficulty level while simultaneously interacting with participants and providing real-time feedback. As presented in the previous sections, we were able to see the differences in the performance measures and gaze data captured by InViRS, which characterize the discriminating gaze behaviors between autistic participants and TD participants. We compared the results between children with ASD and the TD children to establish any meaningful differences in the performance and gaze patterns. Our findings that the children with ASD exhibit atypical gaze patterns are consistent with other works on gaze related study of autistic individuals [3], [4], [44], [46]. For examples, in our study we found that children with ASD had lower ratio of fixation on eye compared to other facial features which was consistent with what was observed in [4], and they took longer time to respond to gaze prompts that was also found in [44], [46].

Although the results discussed above show promise, it is important to highlight the limitations of the study and important targets for future research. First, it was a short study with a relatively small sample size. A longitudinal study with a larger sample size would enable more complex analyses of InViRS’s assistive capabilities and its impact. However, we believe that these preliminary results provide motivation and justification for a resource-intensive longitudinal study in the future. Next, there was no control group for this study. While it is not uncommon to not have a control group for a preliminary evaluation of a new system, we plan to include a control group in our future study to further assess the impact of InViRS in improving joint attention. Additionally, it will be interesting to explore the use of different facial expressions in RJA and its effect on children with ASD for joint attention tasks. It will also be beneficial to evaluate system functionality across different game types other than the two types of games we have used in this work. Finally, generalizability of the skills learnt in InViRS needs to be demonstrated in real-world situations. However, despite these limitations, results from the pilot study showed the potential of InViRS in improving both gaze sharing and gaze following skills in children with ASD. To our knowledge, this is the first such system and study that systematically manipulated these important components of joint attention skill. In addition, InViRS allowed measurement of several quantitative task-relevant metrics and provided real-time feedback to the participants to help them work on their RJA skills.

## Figures and Tables

**TABLE I:** FSM Tuple

Tuple	Definition	Bubble Popping game	Tangram Puzzle game
Q	set of states	{Initialize, Avatar Prompt, Bubble Pop}	{Initialize, Play Avatar, Show Puzzle Color, Enable Puzzle Movement}
Σ	set of inputs	{gaze, complete}	{gaze, mouse, complete}
q_0_	initial state	Initialize	Initialize
F	set of final states	Initialize	Initialize

**TABLE II:** Different Levels of Avatar Gaze Prompts

Game Number	Avatar gaze prompt level
1, 2, 3	Head movement + Full eye movement
4, 5, 6	Full eye movement
7, 8, 9	Minimal eye movement

**TABLE III:** Assistive Prompts in Tangram Puzzle Game

No. of Attempts	Assistive Prompts	Reason for assistance
0	(1) Highlight avatar’s eye region	Initial condition
1	(1) Highlight avatar’s eye region + (2) Sound cue	Participant did not make eye contact with the avatar
2	(1) Avatar repeats gaze prompt at a lower speed	Participant did not select the correct game object
3	(1) Avatar repeats gaze prompt at a lower speed + (2) Highlight the game object + (3) Rotating game object in place	Participant did not select the correct game object
>3	(1) Avatar automatically moves the game object to the target location	Participant did not select the correct game object

**TABLE IV:** Characteristics of Participants

Participants	ASD (n = 9)	TD (n = 9)

	Mean (SD)	Mean (SD)
**Age**	11.00 (1.35)	10.98 (1.98)
**Gender (% male)**	55.6 %	55.6 %
**SCQ Lifetime Total Score**	21.56 (7.33)	2.33 (2.69)
**SRS-2 Total Score**	101.78 (18.54)	24.00 (27.06)
**SRS-2 T-score**	78.22 (7.38)	48.44 (16.12)

SRS-2: Social Responsiveness Scale, Second Edition SCQ: social communication Questionnaire

**TABLE V:** List of Performance Metrics

Performance Metric	Description
Score	One point is received when a participant looked at the correct game object (i.e., a target bubble) that was prompted by the avatar. Maximum possible score is 50.
Time to complete (seconds)	Total time it takes by a participant to interact with the avatar and selecting the bubble for all 50 gaze prompts. Game is terminated if 120 seconds pass by without any interaction by the participant at all.
Response time (seconds)	Response time is computed between the time when the avatar provides a gaze prompt and the time the participant looks at the correct bubble. The time is reset when no gaze interaction is detected after 30 seconds. After that time, the avatar provides a new gaze prompt and the timer starts again.
Fixation points	Gaze fixation was calculated using EyeMMV toolkit [40] in MATLAB based on ROIs parameters; i) name of the ROIs and ii) duration of gaze on ROIs. (Figure 4 illustrates all the facial ROIs)
Ratio of gaze fixation on eye to gaze fixation on other facial features	Ratio of number of gaze fixation points on the avatar’s eye region compared to number of gaze fixation points on other facial ROIs

**TABLE VI:** Overall Performance Measures Results

Participants		Pre	Post	T-test	

		Mean (SD)	Mean (SD)	*p-value*	|*d*|
**ASD**	**Highest score**	38.56 (16.82)	46.89 (5.06)	0.1313	**0.6711**
	**Time to complete (seconds)**	244.04 (74.74)	164.18 (39.93)	*0.0106	*1.333
	**Response time (seconds)**	3.44 (2.98)	1.72 (0.91)	0.0922	**0.7789**

**TD**	**Highest score**	47.56 (3.78)	48.67 (2.24)	0.2145	0.3579
	**Time to complete (seconds)**	192.90 (128.99)	169.67 (90.34)	0.32	0.2086
	**Response time (seconds)**	1.63 (0.76)	1.20 (0.48)	0.0608	**0.6702**

**TABLE VII:** Game Score Measures Based on Speed Groups

Speed Group	ASD		TD	

	Pre	Post	Pre	Post
**Group 1**	9.89 (0.33)	10.00 (0)	10.00 (0)	10.00 (0)
**Group 2**	8.00 (4.00)	10.00 (0)	10.00 (0)	10.00 (0)
**Group 3**	7.78 (4.41)	10.00 (0)	10.00 (0)	10.00 (0)
**Group 4**	7.78 (4.41)	9.56 (1.33)	10.00 (0)	10.00 (0)
**Group 5**	5.89 (4.48)	7.78 (3.56)	7.78 (3.67)	8.89 (1.96)

**T-test**	***p-value***	*0.0139	***p-value***	0.3739
	|***d***|	*1.6050	|***d***|	**0.5200**

**TABLE VIII:** Results for Gaze Fixations on Avatar’s Face

Participants		Pre	Post	T-test	

		Mean (SD)	Mean (SD)	*p-value*	|*d*|
**ASD**	**Total Face Fixation**	160.33 (46.29)	119.22 (46.95)	*0.0056	*0.8914
	**Normalized Eye Fixation**	0.42 (0.25)	0.60 (0.15)	^1^0.6546	^1^0.2688
	**Normalized Other Facial Features Fixation**	0.58 (0.25)	0.40 (0.15)	^2^*0.0266	^2^*1.0474

**TD**	**Total Face Fixation**	139.33 (104.66)	131.78 (74.76)	0.6700	0.0830
	**Normalized Eye Fixation**	0.63 (0.22)	0.59 (0.24)	^1^0.1876	^1^0.3556
	**Normalized Other Facial Features Fixation**	0.37 (0.22)	0.66 (0.24)	^2^0.8766	^2^0.0267

1 p-value and Cohen’s D value calculated using actual fixation points on avatar’s eye region

2 p-value and Cohen’s D value calculated using actual fixation points on avatar’s other facial features
